# Regulatory T-Cells in Pregnancy: Historical Perspective, State of the Art, and Burning Questions

**DOI:** 10.3389/fimmu.2014.00389

**Published:** 2014-08-21

**Authors:** Maria Grazia Ruocco, Gérard Chaouat, Laura Florez, Armand Bensussan, David Klatzmann

**Affiliations:** ^1^Sorbonne Université, UPMC Univ Paris 06, UMRS 959, Immunology-Immunopathology-Immunotherapy (I3), Paris, France; ^2^INSERM, UMRS 959, Immunology-Immunopathology-Immunotherapy (I3), Paris, France; ^3^INSERM, U 976, Hôpital Saint Louis, Paris, France; ^4^AP-HP, Hôpital Pitié-Salpêtrière, Biotherapy (CIC-BTi) and Inflammation-Immunopathology-Biotherapy Department (i2B), Paris, France

**Keywords:** suppressor T-cells, NK cells, cancer tolerance, Treg, evolution of the immune system

## Abstract

In this review, we first revisit the original concept of “suppressor T-cells” in pregnancy, put it in a historical perspective, and then highlight the main data that licensed its resurrection and revision into the concept of “regulatory T-cells” (Tregs) in pregnancy. We review the evidence for a major role of Tregs in murine and human pregnancy and discuss Treg interactions with dendritic and uterine natural killer cells, other players of maternal–fetal tolerance. Finally, we highlight what we consider as the most important questions in the field.

## On the Rise and Fall of Suppressor T-Cells in (Reproductive) Immunology

The history of immunosuppression in pregnancy started in the 1970s, just after the discoveries of Gershon and Kondo (1), when transplantation and tumor immunologists devoted much work to suppressor T-cells (Ts) and suppression. Since 1953, pregnancy has been viewed as “Nature’s allograft” (2), the maternal immune system being in direct contact with a semi-allogenic organism, deeply engrafted and invasive, without, however, any sign of rejection. Medawar conceived three possible explanations for such a paradox: (i) the uterus is an immunologically privileged site, (ii) the fetus is an antigenically immature body, and (iii) there is a non-specific immune depression of the mother, global or at the maternal–fetal interface. As none of these three hypotheses later proved to be correct, the search for an active phenomenon started.

Likewise, as recalled by Trowsdale and Betz (3), the discovery of infectious tolerance by Gershon and Kondo awakened the search for pregnancy-induced Ts. It was first shown that multiple syngeneic pregnancies of C57BL/6 female mice induced tolerance to the male-specific H-Y antigen, as females showed delayed rejection of male skin grafts expressing H-Y (4). A few years later, Simpson et al. reported that multiparity induced a state of tolerance transferable by T-cells (5), and we demonstrated that allo-multiparity evoked systemic T-cell tolerance or hypo-responsiveness to paternal alloantigens (6). Importantly, there were no reports showing that Ts could be involved in tolerance to the first allopregnancy, leaving the question of maternal tolerance to fetal alloantigens unanswered (except for a single unpublished but well-known study by Baines, presented at the 1981 Bannf meeting).

The question of the specificity of a putative suppressive phenomenon (or cells) was of importance, but inadequately answered or even addressed, as Waldmann pointed out: “For example, on the issue of “antigen specificity,” many of the early claims of antigen-specific suppression lacked the discipline cultivated by the classical serologists, in not performing criss-cross experiments. In other words, to claim antigen specificity in a population of cells or extracts thereof, one had to show that A-type T-cells primed to B would suppress responses to B but not C, but also (and critically) that A T-cells primed to C would suppress responses to C and not B. This might easily have misled them into concluding specificity on insufficient data!” (7).

In contrast, in their elegant human studies, Engleman et al. reported that Ts induced by allopregnancy, and their soluble factors, were specific for both stimulator cells and responder cells in mixed lymphocyte reactions (MLRs) (8, 9). They took advantage of the rather rare existence of two multiparous twins, A and A′, married to B and C. Ts from A suppressed an MLR of A and A′ against stimulator lymphocytes from B, but not C; conversely, Ts from A′ suppressed an MLR of A and A′ against stimulator lymphocytes from C, but not B. None of the A or A′ T-cells could suppress B anti-C or C anti-B MLR, nor those of an unrelated E female against B or C.

Despite intense research in the field, the concept of Ts became shaky in the early 1980s, mostly because the absence of a specific marker for Ts prevented study of the functionality of pure populations of cells. The main data supporting the existence of Ts and suppression were for a long time their linkage to an “I–J” or “I–C” sub-region of the class II murine MHC loci, which were supposed to be coding for Ts as well as antigen-specific and non-specific soluble suppressor factors. The “*coup de grace*” to the concept came from the demonstration that these regions did not exist (10, 11). For several years, the concept of suppression became politically incorrect, with very few scientists “saying the *S*… word in public,” to quote Green (12).

Nevertheless, without always explicitly mentioning Ts, several studies continued to point to a form of regulation of maternal immune status by T-cells during pregnancy. Some of them were done in the now classic CBA x DBA/2J murine model of spontaneous immune abortion, in which it was shown pre-immunization with BALB/c splenocytes had a protective effect and was transferable by T-cells (13, 14). To further investigate the mechanisms underlying this protection, nine recombinant inbred strains between BALB/c and DBA/2 were used for pre-immunization. Only three strains behaved as BALB/c. However, when peripheral lymphocytes from pre-immunized CBA females were used as putative regulatory cells in a CBA anti-BALB/c MLR, there was no correlation between the presence of “suppression” and abortion rates, suggesting that local intrauterine immunoregulation is the determinant of success or failure of allopregnancy (15). Immunoregulation was also supported by (i) reports that hypo-responsiveness or tolerance to paternal antigens was repeatedly demonstrated in multiple allopregnancy, with several studies pointing to an important role of the seminal plasma (16–19) and (ii) the “Th1/Th2” paradigm, e.g., a dominance of the production of Th2 cytokines by the pregnant CBA/J (20–22) as well as the earlier demonstration that in “responder” mice the allopregnancy-induced anti-paternal alloantibody response is dominated by IgG1 (23).

## On the Rebirth of Suppressor T-Cells as Just “Regulatory” T-Cells

In 1995, Sakaguchi and colleagues showed that elimination of CD25^+^CD4^+^ T-cells elicits multi-organ autoimmunity, which could be prevented by reinjection of the same cells (24, 25). These properties would qualify CD25^+^CD4^+^ as Ts. Yet, the trauma induced by the I–J story was probably so strong that they were given the more “benign” denomination of regulatory T-cells (Tregs), though they were cells endowed with suppressive activity.

The presence of the CD25 marker on the surface of these cells enabled their negative or positive selection, and thus demonstration of their suppressive activity in various *in vivo* and *in vitro* settings. However, as CD25 is not only constitutively expressed by Tregs, but is also transiently expressed on activated T-cells, another quantum leap for the biology of Tregs was the discovery of a more specific marker, Foxp3, the master regulator of Treg development and function (26). The understanding that mice and human beings with a genetic defect in Foxp3 developed multi-organ autoimmune diseases (27) sealed the case for the discovery of the long-sought suppressor cells of immune responses.

## On Regulatory T-Cells and Maternal–Fetal Tolerance in Mice

### Treg depletion induces abortion in murine pregnancy

These discoveries impacted reproductive biology, with the resurrection of the concept of T-cell-dependent immunoregulation. We now know that Tregs are rapidly recruited to uterus-draining lymph nodes and activated during the first day after embryo implantation (28). These Tregs have the phenotype of activated/memory Treg subsets and are, at least in part, self-Ag specific (28).

The functional importance of this recruitment has been highlighted by transfer/depletion experiments. Aluvihare et al. first noted that Tregs increased markedly in all lymphoid organs of C57BL/6 females mated with CBA males. Importantly, a similar increase was observed whether syngeneic or allogeneic matings were performed, suggesting that this was an alloantigen-independent phenomenon. The cells obtained from B6 mice allopregnant of CBA were able to suppress *in vitro* an MLR of B6 responder T-cells stimulated by CBA cells. However, and rather surprisingly, third party stimulators, MLRs, were not tested for sensitivity to suppression. The authors also transferred lymphocytes from BALB/c females, either allopregnant from a C57BL/6 male or syn-pregnant, into a nude BALB/c mouse subsequently mated with a C57BL/6 male. Such a pregnancy proceeded normally if the whole lymphocyte population was transferred, but the transfer of lymphocytes depleted of CD25^+^ cells resulted in a high rate of fetal resorptions, and T-cells massively infiltrated the implantation sites. Interestingly, (i) both T-cells from syn- and allopregnant mice were abortifacient for allopregnancy when depleted of CD25^+^ T-cells and (ii) none of these two CD25-depleted populations caused pregnancy problems in BALB/c syngeneic matings (29). These results indicate that allospecific effector T-cells are responsible for fetal rejection, but also that these allospecific effector T-cells do not require prior exposure to MHC. Importantly, it should be noted that the experimental setting is based on the transfer of T-cells into a lymphopenic mouse, devoid of B- and T-cells. This induces a major non-specific homeostatic proliferation and activation of the transferred T-cells, and thus the setting does not fully reflect immune regulation during physiological pregnancy.

We demonstrated that Tregs are involved in maternal–fetal tolerance using a more physiological setting by directly depleting/inhibiting CD25^+^ cells *in vivo* in pregnant mice, without any further cell manipulation (30). We showed that treatment with anti-CD25 antibodies did not affect syn-pregnant BALB/c mice, but induced fetus resorption in BALB/c allopregnant females. Incidentally, it should be noted that in all the experiments reported, it was not tested whether elimination of Tregs affected primarily, or exclusively, male (H-Y^+^) fetuses – see Kahn and Baltimore (31).

### Treg expansion/activation or transfer reduces abortion in murine pregnancy

Zenclussen and co-workers have extensively used the CBA x DBA/2J model of naturally occurring murine spontaneous abortion (32–35), initially described by us in 1983 (13). The authors claimed that they were able to “completely prevent” abortion in CBA x DBA/2J mice by transferring Tregs from alloimmunized mice, reporting also “no abortion” at all in the controls CBA x BALB/c and CBA x CBA (32–34). They also deduced antigen specificity from the “complete protection against abortion” (0%) obtained by transferring Tregs from BALB/c-mated CBA/J females, but not those from C57/BL6-mated CBA/J females. Furthermore, transfer of Tregs from the CBA/J x CBA/J mating combination was also protective, which is rather surprising in terms of antigen specificity (35). These results are puzzling since every mammal species (murine strains included) have a strain-specific abortion rate (see, for example, the records of the Jackson laboratory), depending notably on genetic chromosome anomalies, most of them occurring as a consequence of meiosis.

More recently, the same authors showed that Treg-transferred CBA/J females treated with anti-IL-10 – but not anti-TGF-ß – prior to mating with DBA/2J males had an increased abortion rate (36). In this line, we have reported that anti-IL-10 treatment selectively affects CBA x DBA/2J mating, but not other mating combinations (22).

We investigated whether *in vivo* Treg expansion/activation could improve successful pregnancy rates. We observed that Treg stimulation, either directly by low-dose IL-2 or indirectly by Fms-related tyrosine kinase 3 ligand, led to normal pregnancy rates in CBA x DBA/2J abortion-prone mice (28).

Conversely, high doses of intravaginal interferon have been shown to be abortifacient and/or anti-implantation not only because of their classic effects in conjunction with TNF but also by reducing Tregs and IL-17 at the implantation site (37).

### Treg changes during the estrous cycle

A further case for an important role of Tregs in pregnancy is the observation that the uterus “prepares” itself for pregnancy by specific cyclic accumulation of Tregs (38, 39).

Kallikourdis et al. studied changes in the numbers of T-cells in the uterus together with the expression levels of chemokines known to induce Treg migration. A rise for CCL3, CCL4, CCL22, and CX3CL1 was noted from diestrus to estrus. If mating led to pregnancy, only CCL4 remained high. In fact, there was a direct correlation between uterine CCL4 expression and Foxp3^+^ T-cells. Moreover, from estrus to gravid uterus, CCR5^+^ cells rose from 50% to more than 70%. The authors concluded that since “alloantigen-experienced effector Tregs” express CCR5, CCL4 might be responsible for the retention of these cells in the gravid uterus (39).

Hormonal changes may be drivers for Treg changes. In particular, estrogen has been shown to induce expansion of Foxp3^+^ cells (40, 41), including in the (pregnant) uterus (42). Analyses of Treg suppressive activity in wild-type, estrogen receptor knockout (ERKO), and programed death-1 (PD-1) KO mice, revealed that (i) estrogen induces PD-1 in CD4^+^Foxp3^+^ cells and (ii) PD-1 expression as well as Treg suppressive activity was reduced in estrogen receptor KO mice. Pre-treatment of PD-1 KO mice with estrogen led to a partial recovery of Treg suppression without enhancing Foxp3 expression. Yet, PD-1 is likely not the only pathway controlling Treg activity, since Treg function is also partly restored by estrogen in PD-1 deficient animals (43). Thus, both PD-1-dependent and PD-1-independent pathways could be involved in estrogen-mediated Treg suppressive activity. Estrogen has also been shown to directly influence Treg expression of IL-10 (44).

An increase of Tregs in mice at day 2 of pregnancy has been described, except for the CBA x DBA/2J mating combination (45), which led to the conclusion that Tregs do not depend on hormonal levels. This is in disagreement not only with the aforementioned reports but also with the data of Mao et al., who showed an increase in Tregs in mid-pregnancy, which is at least in part progesterone-dependent and correlates with an increase in IL-10 production by Tregs (46).

The human chorionic gonadotropin (hCG) has been reported to attract Tregs locally in the murine uterus (47, 48). Similarly, as mentioned before, the luteinizing hormone (LH) has been reported to completely prevent abortion in the classic CBA x DBA/2J murine model of immune abortion, which correlated with increased Treg numbers both locally and at the periphery (45).

### The influence of mating/seminal fluid on Tregs

Events occurring early during pregnancy seem to influence future Treg expansion/function. Using several murine models, Robertson’s group demonstrated that mating itself is important for successful pregnancies, with the seminal plasma driving the immediate and preparing the future expansion of uterine and, likely, systemic Tregs. This induces a (transient) “tolerance-like” state to paternal alloantigens in mice. Moreover, the authors showed that seminal fluid contains both TGF-β and prostaglandin E, which potently induces Tregs (49, 50).

## On Regulatory T-Cells in Human Pregnancy

In human beings, Saito’s group identified decidual Foxp3^+^ Tregs in uterine biopsies (51, 52). Robertson’s group showed the presence of Foxp3 mRNA in the uterus of normal women by qRT-PCR in endometrial biopsies obtained during the mid-secretory phase of the menstrual cycle. Interestingly, they found that Foxp3 mRNA levels decrease two-fold in patients with primary unexplained infertility compared with fertile women (53). However, they could not correlate this result with endometrial cytokine levels (TGF-β1, TGF-β2, TGF-β3, IFN-γ, IL-2, IL-4, IL-5, IL-10 and IL-12p40, IL-1α, IL-1β, IL-6, LIF, GM-CSF, and TNF-α) (53).

Fainboim and colleagues monitored Tregs in the menstrual cycle of fertile and infertile women (54) and showed a periodic modulation of Tregs. Treg levels peaked in the late follicular phase, which correlated with serum estradiol, and decreased markedly in the luteal phase. Interestingly, they also showed that in patients with recurrent spontaneous abortions (RSAs), Tregs were low and changes in Treg numbers in the follicular or luteal phase were not significant. Treg numbers in women with RSAs were very similar to the numbers observed in post-menopausal women (54). Furthermore, when these Tregs were tested for their suppressive capacity, a higher number of cells was required to obtain the same level of suppression as Tregs from fertile women, suggesting that, in RSA patients, Tregs are functionally defective (54).

Likewise in mice, the influx of Tregs in the decidua is not only dependent on the hormonal levels in the environment but is also linked to the intercourse, which temporarily increases their number (50). In RSA, the reduction of Tregs appears not to be related to the reduced levels of IL-6 and rIL-1α mRNAs. On the contrary, the relative abundance of mRNAs encoding for LIF, GM-CSF, IFN-γ, IL-1β, IL-4, IL-5, IL-10, IL-12p40, TNF-α, TGF-β1, TGF-β2, and TGF-β3 remained unaltered regardless of the fertility status (53, 55). In this context, IL-27 has recently been suggested to regulate Tregs, IL-17, and IL-10 expression (56).

Besides hormones and cytokines, trophoblasts can also recruit and induce Tregs. The high levels of TGF-β produced by trophoblasts both induce and recruit CD4^+^ peripheral Tregs (pTregs) *in vitro*. Trophoblasts can also activate some CD8^+^ regulatory cells, which are independent of MHC class I, have a restricted TCR repertoire, and co-express the mucosal markers CD103 and CD101 (57). In the blood of pregnant women, they rapidly expand, suggesting a potential role for these cells *in vivo*. Despite extensive evidence of their role in regulating immune responses – see for example (58–60) – the role of the CD8^+^ Treg subset in pregnancy or embryo implantation is still poorly understood.

Regulatory T-cells may also be involved in pre-eclampsia (PE), together with regulatory NK T-cells (52, 61, 70, 71), as reported by several authors (62–65), except for Paeschke et al. (66). Furthermore, it has been suggested that there might be an imbalance between CD4^+^CD25^hi^Foxp3^+^ and CD4^+^CD25^−^Foxp3^+^ Treg subsets in PE (61). Recently, however, not only Tregs but also HLA-G^+^CD4^+^ T-cells have been suggested to play a role (65).

## On Regulatory T-Cell Specificity in Pregnancy

The studies discussed so far point to an interesting problem: what is the specificity of Tregs mobilized for successful pregnancy? This question was recently addressed by Rowe and colleagues, who showed that pregnancy primes the selective accumulation and activation of maternal Tregs with fetal specificity (67). The authors employed transgenic mice that expressed a surrogate fetal antigen, the I-A^b^ 2W1S_55–68_ peptide. They found that pregnancy-induced maternal CD4^+^Foxp3^+^ cells specific for I-A^b^ 2W1S_55–68_, a peptide that expressed CD44 and rapidly accumulated during mid-gestation. These cells persisted at levels increased approximately 10-fold through day 100 post-partum. The same maternal Tregs with fetal specificity expanded at an accelerated rate during secondary pregnancy with the same partner. Using the Foxp3-DTR model (68), the authors also demonstrated that the expanded cells were pTregs and that partial ablation of Tregs in Foxp3-DTR/WT mice resulted in reduced fetal abortion rates compared with primary pregnancy (67).

In pregnant mice, a reduced number of paternal antigen-specific T-cells (69), likely due to peripheral clonal deletion (70), and a reduced responsiveness to tumors bearing the same paternal antigen (T-cell awareness of pregnancy), was demonstrated using a transgenic mouse model and a weakly antigenic tumor allograft challenge. This was interpreted as implying that multiple tolerogenic mechanisms are at play at the same time. T-cell phenotype and responsiveness to tumors was restored after delivery (69).

This questions the antigen specificity of pregnancy-induced Tregs. In the aforementioned system, it has been shown using MHC tetramer that Tregs are not themselves Ag specific, but mediate antigen specificity by locally anergizing the highly specific effector T-cells (67, 69–73).

However, the existence of “true” antigen specificity of Tregs involved in maternal–fetal tolerance is claimed in several studies in human beings (54, 74) and in mice (31, 35, 36, 75). In the classic CBA x DBA/2J murine resorption model, Treg function has been shown to be elicited by the paternal-specific “protective” peptide (76). Similarly, the data of Kahn and Baltimore in an elegant transgenic system support specificity in the regulation of anti H-Y responses (31).

In contrast, we find in the very same model that Treg expansion is driven, at least in part, by recognition of self-specific antigens (28).

This apparent contradiction could be solved if in the uterus and draining lymph nodes two different Treg subsets were mobilized at implantation and later throughout pregnancy, one being self-specific, the other being fetus-Ag or MHC-specific. As reviewed by Marrack et al. in “T-cells and their eons-old obsession with MHC” (77), T-cells could be both antigen- and MHC-specific and thus self-biased. The different loops created by the germline-encoded and non-germline portions of the TCR may contact the MHC proteins and the peptide bound on the MHC, respectively. This idea comes from the observation that there are many TCR variable elements that form specific patterns to contact a particular site on the MHC. Mutations in these sites affect the ability of T-cells to react with the MHC. Interestingly, these similar elements were found in evolutionarily distant species, such as sharks and human beings, suggesting that they evolved to allow TCR to react with MHC proteins.

## On Regulatory T-Cells in Evolution: The Development of Thymic and Peripheral Tregs and Their Role in Maternal–Fetal Tolerance

Placentae appeared very early, and reappeared at various stages during evolution. Velvet worms – onychophora – are placental viviparous, as are sharks and other fishes, some dinosaurs and reptiles, too. Placentation in eutherian mammals came later as the first mammals, the monotremes, are oviparous. The placental mammals emerged 165–80 million years ago (the oldest known eutherian fossil so far being 160 million years old, the *Juramaia sinensis* (78), which fits with most DNA clock analysis of the separation between eutherians and marsupials. The first well-documented placental eutherian is the 65-million-year-old *Maelestes gobiensis* (79).

The first viviparous mammals, in between dinosaurs and mammals, were faced with the development of a sophisticated adaptive immune system, a challenge not previously present. Marsupials escaped the threat of fetus rejection just before it appeared by using the marsupial pouch to house the quasi-fetus newborn. The development of placentation in eutherians involved a series of suppressive mechanisms. Only a few of them have been demonstrated to be crucial, including those involving Tregs.

Thymic Tregs (tTregs) differentiate in the thymus following up-regulation of Foxp3 as a consequence of their expression of self-antigens highly reactive TCRs. pTregs generate in the periphery upon stimulation with high-affinity cognate TCR ligands in the presence of TGF-β and retinoic acid (80–83). The observation that CNS1 – an intronic *Foxp3* enhancer containing Smad3 – and retinoic acid receptor (RAR)-binding sites facilitate TGF-β-dependent Foxp3 induction and pTreg cell differentiation, but is dispensable for tTreg generation, suggests that the biological functions of these two Treg cell subsets are distinct (84).

Samstein and co-workers generated CNS1-deficient mice (85), which lack only pTreg cells but not tTregs. They observed that pregnancy in these mice resulted in a high abortion rate in allogeneic, but not syngeneic matings. Moreover, ablating tTregs in the CNS1-deficient mice did not enhance allopregnancy abortion (85). Hence, they concluded that pTregs are necessary for successful pregnancy while tTregs are dispensable.

The CNS1 non-coding sequence does not exist in other phyla, such as non-mammals, and in mammals is present only in eutherians, but not in marsupials. Thus, Samstein and co-workers concluded that “the mechanism of extrathymic differentiation of pTreg cells may have been gained during evolution to reinforce tolerance to paternal alloantigens presented by the fetus during the increasingly long gestation period in placental mammals” (85). However, the authors also reported a defect in spiral artery formation in mice lacking pTregs, which open other possibility than just tolerance for the role of pTregs in pregnancy.

We believe that the unique role of pTregs should be balanced by the fact that there exist yet no models of a pure tTreg depletion, which could demonstrate the role – or absence of role – of this subset in maternal–fetal tolerance. Furthermore, two gestational periods should be considered: the embryo implantation period and later fetus development. We showed that the immediate response of the immune system to embryo implantation is mediated by activated/memory self-specific Tregs, hence tTregs. It is thus possible that tTregs initiate a tolerance state that is later maintained with the recruitment of pTregs. We believe that both tTregs and pTregs have been selected during evolution primarily for the purpose of establishing maternal–fetal tolerance in eutherians (28, 85).

## On Similarities between Regulatory T-Cell Responses to Fetal and Tumor Growth

As often mentioned in the literature since the dawn of Reproductive Immunology, there are striking similarities between malignant processes and pregnancy (86). In a tumor model, we observed that tumor emergence elicits a brisk Treg response that precedes and preempts the response of effector T-cells. This Treg response is detectable as soon as days 2–3 post-tumor cell implantation or emergence and is mediated by self-antigen-specific CD44^hi^CD62^low^ activated/memory Tregs (87). We recently reported striking similarities in the Treg response to embryo implantation, with the same recruitment of self-antigen-specific CD44^hi^CD62^low^ activated/memory Tregs detectable within 2 days post-implantation (28).

However, the parallel is not complete. Pre-immunization against an artificial paternal antigen (HA in our case) only marginally increased fetal loss, whereas pre-immunization with the HA antigen resulted in 100% eradication of HA-expressing tumors. However, mixing Treg depletion with pre-immunization drastically increased fetal loss (28).

Furthermore, the immunological paradox of pregnancy, whereby the maternal immune system tolerates the presence of the semi-allogenic fetus, has historically been associated with the early work on immunological tolerance to transplantation. However, even though Tregs play a role in the control of allogeneic responses to solid or cell grafts (including allogeneic cancer cells) (88) and have demonstrated therapeutic potential in this setting (89), these grafts are always rejected in the absence of specific intervention. This highlights the uniqueness of the immune responses in the allogeneic maternal/fetal tolerance setting.

We hypothesized that the similarities in the Treg response to tumor or embryo implantation suggest that protection of cancer cells by Tregs became the price paid for an efficient protection of embryos (28).

## On Other Important Cells

The decidua is populated by several immune cell types, which coexist together with stroma cells and trophoblasts. Among them, dendritic cells (DCs) and uterine NK (uNK) cells are highly abundant. During the female estrus cycle and throughout pregnancy, the number of these cells undergoes dramatic chances, as do, likely, their reciprocal interactions. The concept of decidual cell–cell interactions is relatively new and arises from an important feature of immune cells, their ability to migrate, which confers them dynamic properties. The introduction of intravital two-photon microscopy made it possible to study the dynamic behavior of immune cells and their interactions, in a spatio-temporal dimension. However, information about immune cell dynamics at the maternal–fetal interface remains limited, while abundant in other models such as cancer, infection, or inflammation [reviewed in Ref. (90)]. T-cells are relatively rare in the uterus of both pregnant and non-pregnant human beings and mice (91, 92). However, despite their paucity, Tregs are critical for normal pregnancy. The secret of their pivotal role could thus reside in the dynamic interactions they establish within the decidua.

### Uterine NK cells

Uterine NK have long been considered the most important cell type for the success of pregnancy due to their abundance in the decidua. Moreover, as increasing evidence points to the importance of other leukocytes, such as Tregs, the functional relationship between uNK cells and the other immune cells has come into focus.

Uterine NK cells differ from NK cells in other sites of the body. Mature uNK contain numerous granules (rich in perforin, granzymes, granulysis) (93), but, unlike peripheral blood NK cells, uNK cells are only weakly cytotoxic *in vitro* and do not kill trophoblasts *in vivo*. They seem to both differentiate and proliferate in the uterus, but also migrate from the periphery (94, 95). In mice, uNK increases upon implantation in concomitance with trophoblast invasion of the endometrium and subsequent decidualization (96) and they peak at mid-gestation. Mouse uNK cells have been shown to localize in the mesometrium to form a characteristic ring-shaped structure around the spiral arteries characterized by the presence of highly proliferative cells, called the mesometrial leukocyte aggregate of pregnancy, MLAp (97). Even though models of artificial decidualization have shown that uNK differentiation depends on hormonal changes (98), rather than on trophoblast invasion, mouse uNK cells do not express progesterone receptor (96).

Early implantation sites in mice deficient for NK, T-, and B-cells showed abnormal decidual and mid-gestational myometrial structures and no spiral artery modifications (99–101). Noteworthy, despite these defects, litters of normal size were born (102–105), except in the Tge26 (100, 101) mice, which display a reproductive deficit. Bone marrow transplantation of NK^+^ T^−^B^−^pools before mating restored the defects suggesting a major role of uNK cells more related to vascularization than to tolerance.

In this line, human pregnancy-associated disorders, such as PE, still birth, and fetal growth restriction, all display deficits in spiral artery formation and are characterized by a “shallow invasion” of the uterine wall (106, 107). uNK cells produce several angiogenic factors (108–110) such as IFN-γ, and Croy and colleagues have shown that artery remodeling is strictly dependent on IFN-γ produced by NK cells in the uterus (111, 112). However, in human beings, the levels of IFN-γ during pregnancy are rather low although spiral artery remodeling remains crucial (113, 114). Trophoblasts express a characteristic combination of HLA-C, HLA-G, and HLA-E MHC class I molecules (115) and correct spiral artery remodeling has been correlated to allo-recognition of trophoblasts cells by uNKs.

### Uterine dendritic cells

Together with uNK cells, DCs represent the most abundant cell type in the uterus. They are known as potent antigen-presenting cells (APC). DCs have been reported to recognize foreign antigens present on sperm cells upon mating (49, 50, 116), but most likely also recognize alloantigens expressed by the invasive trophoblasts during implantation and decidualization. Interestingly, upon implantation, DCs re-localize to different areas of the decidua (117). In particular, Erlebacher and co-workers have suggested that the decidua works as a barrier that impedes DCs to efficiently prime T-cells in the lymphoid organs, to minimize the immune response to paternal alloantigens (118). Furthermore, they described how DCs remain entrapped in the uterus and are unable to carry antigens to the lymph nodes due to the lack of lymphatic vessels, which, in the mouse uterus, are confined exclusively to the myometrium (118, 119). Importantly, they demonstrated the spatio-temporal regulation and the extent of antigen-specific T-cell priming during pregnancy (120). They mated wild-type females with Act-mOVA males, where OVA expressed by the conceptus mimics a paternal-specific antigen. Only DCs of maternal origins presented OVA-MHC in the LNs and induced OVA-specific T-cell expansion at mid-gestation, suggesting that the LNs are the primary site of alloantigen-presentation.

Finally, uterine DCs have also been proposed to perform trophic functions. Plaks and co-authors induced fetal loss by depleting DCs before implantation (121) using the CD11c-DTR transgenic mouse model (122). The absence of DC-derived angiogenic factors hampered vessels’ formation, and affected normal implantation and decidualization. Taken together, these results indicate that during mouse pregnancy, DCs prime T-cells and play a trophic function by ensuring correct vessel formation.

### On Treg cross-talks

Similar to Tregs, uNK cell numbers vary during the estrus cycle. Recent results from Rudensky’s group have highlighted a defect in spiral artery formation in mice lacking pTregs (85). Absence of pTregs determines fetal demise in their model. These results pose an interesting question: is there co-operation between uNK cells and Tregs to ensure correct spiral artery modification?

Moreover, Rowe and colleagues have recently shown that maternal Tregs specific for paternal alloantigens expand > 100 folds during pregnancy (67). These cells persist after delivery and, because of their antigen-specific memory, expand faster than naïve Tregs in subsequent pregnancies, possibly contributing the well-known “lymphoid recall flare” in second pregnancy (123). Relating these results to the human situation, the authors suggest that their observations might explain why the rates of pregnancy complications, such as PE, decrease in subsequent pregnancies. Taken together, both in human beings and mouse, uNK cells and Tregs seem to affect spiral artery formation with important consequences for fetal survival. Moreover, Tregs can also suppress NK cells. uNK cells in turn might control Treg recruitment to the pregnant uterus.

Furthermore, DC maturation in the pregnant uterus is thought to support expansion of antigen-specific Tregs that finally protect the fetus from abortion (49). Thus, DCs have been proposed to exert a dual role in promoting tolerance to paternal alloantigens, limiting their own priming-activity, also in response to signals in the microenvironment, and priming the few Tregs present in the decidua.

Finally, the known cross-talk between NK cells, DCs, and Tregs may be operating locally in the uterus during pregnancy (124).

## On Burning Questions

**Figure 1:**
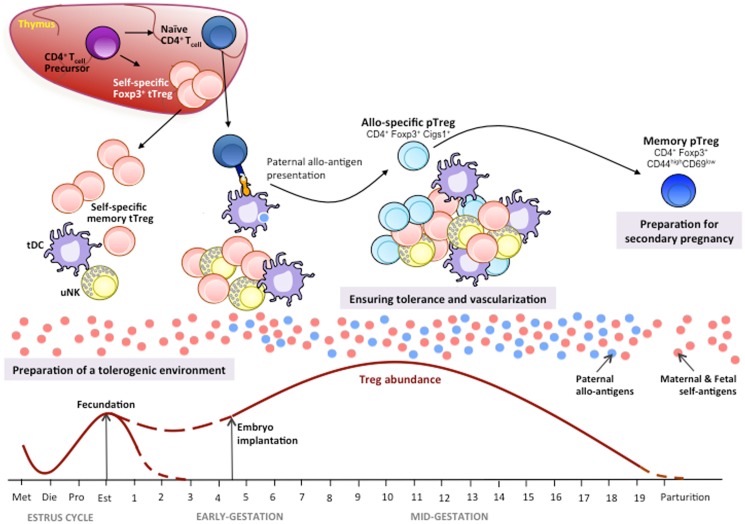
**Tregs in mouse pregnancy**. Thymic Tregs (tTregs) recognizing maternal/fetal self-antigens differentiate in the thymus from the CD4^+^ T-cell precursors by up-regulating Foxp3 expression. During the estrus cycle, there is an increase in tTregs in the periphery and the uterus where, together with tolerogenic dendritic cells (tDC) and uterine NK (uNK) cells, they prepare a uterine tolerogenic environment for pregnancy under a hormonal control. During the estrus phase, recruitment of tTregs at ovulation is maximum in order to prepare a tolerogenic uterine environment for a potential embryo implantation. During pregnancy, self-antigen-specific activated/memory tTregs mount a first-line tolerogenic response (28). Later, the first fetal/paternal alloantigens generated by fetal cells trigger an immune response to paternal alloantigens (85). Alloantigen presentation through tDCs favors the conversion of naïve CD4^+^ T-cells in induced peripheral Tregs (pTregs) by up-regulating Foxp3 and its Cigs1 enhancer gene expression (84). The clonal expansions of allospecific pTregs together with the proliferation of tTregs, uNKs, and tDCs during the mid-gestation periods ensure the maintenance of immune tolerance to the fetus and allow vascularization to guarantee a steady supply of nutrients and oxygen to the fetus for a proper growth and development. Generation of memory pTregs specific for paternal antigens will contribute to tolerance induction to the same fetal/paternal alloantigen exposure in case of a secondary pregnancy with the same paternal antigens. The tTregs, uNKs, and tDCs cross-talk is yet poorly defined.

Since the discovery of Tregs 30 years ago, our knowledge about immune tolerance has dramatically improved. The data summarized above suggest their important role in conserved mechanisms that establish and maintain immune tolerance during early pregnancy (Figure 1).

We believe that most important points for the field that remain unanswered or controversial are: (i) the antigen specificity of Tregs involved, which could be elucidated by TCR deep-sequencing, (ii) the respective functional role of the Treg subsets involved (i.e., tTreg, pTreg, etc.), (iii) the localization and functional cross-talk of Tregs with uNK and uDCs, which could be studied by intravital imaging and novel transgenic mice (125–127), and (iv) the link between Treg responses to embryo and tumor cell implantation.

A better understanding of these mechanisms will be pivotal in identifying more effective therapeutic targets for the treatment of pathological conditions related to pregnancy (128, 129) and, more generally, to diseases in which the immune balance is perturbed.

## Conflict of Interest Statement

The authors declare that the research was conducted in the absence of any commercial or financial relationships that could be construed as a potential conflict of interest.
